# Analysis of Research Productivity of Orthopedic Surgery Residency Applicants

**DOI:** 10.7759/cureus.46384

**Published:** 2023-10-02

**Authors:** Bolatito Adeyeri, Tiffany Lee, Taylor Beal, Austin Huang, Melvyn A Harrington

**Affiliations:** 1 Department of Orthopedics, Baylor College of Medicine, Houston, USA; 2 Department of Ophthalmology, Baylor College of Medicine, Houston, USA

**Keywords:** undergraduate medical education, residency applications, altmetric score, medical student education, publication count, research productivity, orthopedic residency, altmetric attention score

## Abstract

Background

Orthopedic surgery has become an increasingly competitive specialty. With a pass-fail Step 1, an even greater emphasis on research has been placed to allow candidates to better distinguish themselves. This study analyzes the scholarly activity of accepted orthopedic residency applicants during medical school, assessing what factors, including the novel altmetric attention score, may be associated with greater research productivity.

Methods

A list of orthopedic residency programs was obtained from the Electronic Residency Application Service (ERAS). A total of 688 orthopedic residents from 180 programs who matriculated in 2020 from allopathic medical schools were identified. Resident demographic information and bibliometric data (total publications, orthopedic-related publications, h-index, and altmetric score) of publications published from July 1, 2016, to September 1, 2020, were collected. Descriptive statistics were calculated. Kruskal-Wallis tests analyzed the association between medical school characteristics and research productivity using Stata® 17.0 (StataCorp LLC, College Station, Texas).

Results

Postgraduate-Year-3 orthopedic residents (N=688) published 2,600 articles during medical school, averaging 3.8 articles per resident. The residents from a top 25 medical school for research had publication counts, altmetric scores, and h-indices, on average, that were higher than those from non-top 25 medical schools for research. Over 150 residents had no publications, and ~10 residents had more than 30 publications.

Conclusions

The results illustrate that medical school research status influences the research productivity of applicants. Also, given the average number of publications, most research listed on applications are abstracts and presentations. Utilization of the altmetric score may not yet be the best way of examining research experience because orthopedic applicants do not appear to use social networks for academic research.

## Introduction

Orthopedic surgery has become an increasingly competitive specialty, with a match rate of 65.8% in the 2021-2022 cycle [1]. Due to the competitiveness of this field, applicants are advised to find ways to make themselves stand out. According to recent National Resident Matching Program (NRMP) Program Director Surveys, between 50% and 66% of orthopedic surgery residency program directors cite involvement and interest in research as an important factor in deciding applicants to interview [2-4]. Further, with the implementation of a pass-fail Step 1 in January 2022, an even greater emphasis on research may be placed to allow candidates to better distinguish themselves from others [5]. Students are increasingly encouraged to work on more projects or even take a research year to enhance their application [6]. 

The NRMP reports the mean number of Research Experiences and the mean number of abstracts, presentations, and publications (APPs). The NRMP notes that “Abstracts, Presentations, and Publications” include peer-reviewed articles, abstracts, poster sessions, and invited national or regional presentations. However, peer-reviewed articles or publications tend to be emphasized more and are more easily verifiable than posters and abstracts [7,8]. Over the years, the average number of APPs has increased [9]. The mean number of APPs for matched orthopedic applicants has risen from 11.5 to 16.5 between 2018 and 2022. With this rise in reported research productivity, students may attempt to increase the number of papers and projects they involve themselves in for the sake of numbers, rather than participating in more meaningful projects that take longer to yield a publication or poster [7]. And while there is documentation of an upward trend in research endeavors among orthopedic surgery applicants, the quality, caliber, and worth of their publications may not correlate.

Still, there is no consensus on the best way to evaluate both the research quality and quantity of residency applicants. Research involvement, considering only verifiable publications, can generally be characterized by the number of publications, authorship rank, h-indices, journal impact factor, and the impact and quality of the research. The incorporation of the novel altmetric attention score with all these other parameters may give us a more comprehensive assessment of the productivity of orthopedic surgery applicants. The altmetric attention score provides data on how much a scholarly article has been shared or interacted with on social media feeds [10]. This score represents a weighted approximation of the number of mentions on different sites [11]. While the use of social media in orthopedic research has been studied, the altmetric attention score influence, which to date, has not been examined, could be a potential way to analyze research productivity in orthopedic surgery applicants [12]. Often, applicants publish papers in medical school before there would be much time for them to be cited by other authors, while the altmetric attention score highlights more than just citations. 

In this study, we aim to determine whether there are significant differences in research productivity, focusing on publications, among certain groups of residents using parameters such as: attending a US News top 25 medical school for research, attending a US News top 25 medical school for primary care, the number of total papers applicants published in medical school, the number of orthopedic papers published in medical school, the h-index of each resident, and the altmetric attention score of each paper [13,14]. It is possible that medical school research status influences the research publication potential of students and the social media diffusion of their publications. Given that orthopedic surgery residency program directors cite involvement and interest in research as an important factor in deciding which applicants to interview, we seek to verify, at least in part, and demystify the mean number of APPs by examining the number of publications and the possible influential variables. Characterizing and expanding upon the trends in orthopedic surgery residency applicants will better inform residency program directors and medical students in selecting for and becoming the best candidates for match.

## Materials and methods

Study population

A list of orthopedic surgery residency programs participating in the match was obtained from the Electronic Residency Application Service (ERAS) Directory from the Association of American Medical Colleges (AAMC) website. Then, from the website of each residency program, a list of current residents who matriculated in 2020 was compiled to obtain 688 residents from 180 programs (90% of total programs). The matriculation year 2020 was chosen to gather recent data and to ensure all residency programs would have this group of residents listed on their websites. The residents were confirmed using LinkedIn, Doximity, and ResearchGate. Once all the residents for analysis were compiled, a database of demographic information and research productivity, focusing on publications, was created.

Demographic information on each resident included sex, medical school attended, medical school type (allopathic or osteopathic), state of the current residency program, IMG (International Medical Graduate) status, and PhD holder status. Information about the rankings of the medical school they each graduated from, such as if it was considered a Top 25 medical school for primary care or Top 25 medical school for research (according to US News & World Report) was included. US News & World Report criteria for research rankings were calculated from weighted averages of the following components: peer assessment score, residency directors' assessment score, median MCAT score, median undergraduate GPA of the entering class, acceptance rate, faculty resources, total federal research activity, and average federal research activity per faculty member [15]. US News & World Report criteria for primary care rankings were calculated from weighted averages of the following components: peer assessment score, residency directors' assessment score, median MCAT score, median undergraduate GPA of the entering class, acceptance rate, faculty resources, medical school graduates practicing in primary care specialties, and medical school graduates entering primary care residencies [15].

For research productivity, each resident's publication count was collected by first searching each resident in PubMed to analyze their publications from July 1, 2016, through September 1, 2020. These dates were chosen to ensure that only publications produced during their time in medical school were analyzed and not their work done after matriculating into residency. Then, the number of total and orthopedic-related publications for each resident was noted. However, publications listed in PubMed that were erratum, or corrections of errors by the publisher, were not counted as additional publications. The h-index of each author was calculated by determining the number of citations in each publication. The altmetric attention score of each publication was also gathered for each author [16]. The score represents a weighted approximation and not a raw total of the number of mentions the article received [11]. The altmetric attention score is made up of three components: the number of times the article is mentioned, where the mentions came from, and the authors of each mention. The sources are weighted differently and include the following: Twitter, Facebook, Policy documents, News, Blogs, Mendeley, Scopus post-publication peer reviews, Reddit, Wikipedia, Stack Overflow, F1000 reviews, Google+, YouTube, Open Syllabus, and Web of Science [10]. These scores help indicate which research is receiving attention and the extent of that attention relative to its relevant journal. The altmetric attention scores were summed and averaged for each resident.

DO (n = 100), PhD (n = 5), and IMG (n = 9) residents were excluded from the database (Table 1). In a primary survey of the data, we saw that IMG residents tended to have a bimodal distribution for publication counts, either having none (i.e., 0) or many publications (i.e., >40). We also noticed that PhD residents had greater numbers of publications with an average of 19 publications, and DO students had fewer with an average of two publications. Residency programs (n = 16) that did not list their residents on their website were also excluded since we could not reliably confirm their applicable cohort of students for the database. Since the military match does not use ERAS, military programs were also excluded.

**Table 1:** Exclusion criteria IMG: international medical graduate

Criteria	IMG	PhD	DO
Number	9	5	102
Percentage of total residents (out of N = 802)	1.1%	0.62%	12.72%

Statistical analysis

All data were analyzed using Stata® 17.0 (Stata Corp LLC, College Station, Texas). Descriptive statistics were calculated. Kruskal-Wallis tests analyzed the association between medical school characteristics and research productivity. Tests were conducted with and without outliers based on the total number of publications. Outliers (n = 34) were defined as being greater than the 95th percentile of resident statistics and were not included in our analysis. The outliers had more than 14 publications in total.

## Results

Individual research productivity

**Figure 1:**
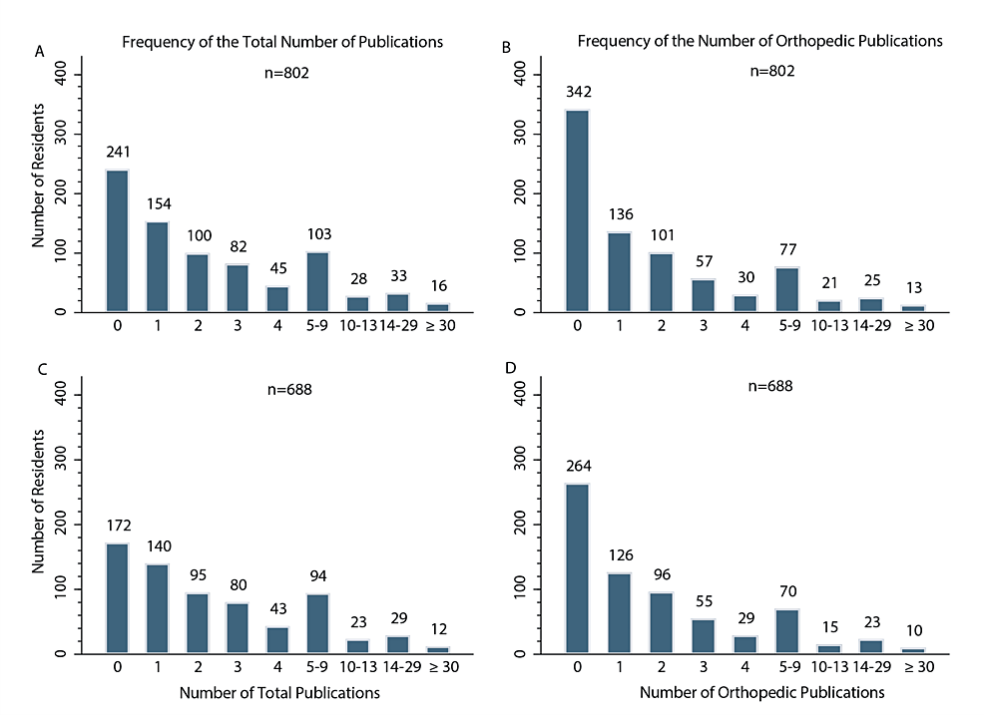
Frequency of publications Frequency of (A) number of total publications among all 802 residents; (B) number of orthopedic publications among all 802 residents; (C) number of total publications among 688 traditional allopathic MD students; and (D) number of orthopedic publications among 688 traditional allopathic MD students.

Postgraduate-Year-3 (PGY-3) orthopedic residents (N = 688) published 2,600 articles during medical school, averaging 3.8 articles per resident (Table 2). The mean total of orthopedic publications was 2.9 per resident. The mean total altmetric attention score (AS) for each resident was 29.4, with each of their articles averaging an AS of 5.1. The mean h-index was 1.8 for each resident. The additional means for these variables taking into consideration the medical school characteristics (female and male residents, residents who attended a top 25 medical school for research and those who did not, residents who attended a top 25 medical school for primary care and those that did not, and those who had a home orthopedic department and those that did not) are listed in Table 2. Additionally, there were over 150 residents who had no publications, and about 10 residents had more than 30 publications (Figure 1).

**Table 2:** Descriptive statistics of medical school research of orthopedic residents *Denotes significance at p < 0.05 in Kruskall-Wallis test

	No. (%, out of 688)	Publications (Mean ± SD)	Ortho Publications (Mean ± SD)	Total AS (Mean ± SD)	Avg AS (Mean ± SD)	h-Index (Mean ± SD)
All Residents	688	3.80±6.81	2.92±6.11	29.42±95.20	5.10±14.68	1.80±3.75
Female	140 (20.35%)	3.16±5.13	2.35±4.82	33.67±119.79	5.90±16.25	1.49±1.79
Male	548 (79.65%)	3.96±7.18	3.06±6.39	28.33±87.92	4.90±14.25	1.89±4.11
Medical School Characteristics						
Top-25 Research*	136 (19.77%)	4.97±6.39	3.90±5.70	50.60±113.10	8.85±19.69	2.76±5.87
Non-Top-25 Research*	552 (80.23%)	3.51±6.89	2.68±6.19	24.20±89.60	4.18±13.01	1.57±2.98
Top-25 Primary Care	133 (19.33%)	3.82±6.38	2.98±6.00	39.77±125.30	6.93±18.13	2.21±5.91
Non-Top-25 Primary Care	555 (80.67%)	3.79±6.92	2.90±6.14	26.93±86.40	4.67±13.70	1.71±3.02
Home Ortho Department	616 (89.53%)	3.74±6.66	2.87±5.88	31.09±99.95	5.41±15.39	1.85±3.93
No Home Ortho Department	72 (10.47%)	4.32±8.06	3.38±7.83	15.08±30.33	2.51±5.00	1.46±1.66

Research productivity as a function of medical school research productivity

**Figure 2:**
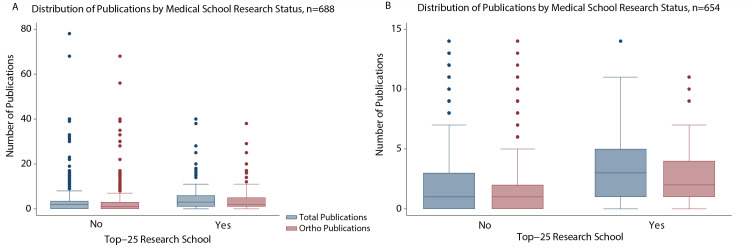
Distribution of publications by medical school research status Boxplots showing that (A) the number of total publications (H(2) = 25.81, *p* < 0.01, η^2 ^= 0.04) and orthopedic publications (H(2) = 24.77, *p* < 0.01, η^2 ^= 0.03) of students from a top 25 research school was greater than those of students from a non-top 25 research school; and (B) the number of total publications (H(2) = 24.48, *p* < 0.01, η^2 ^= 0.03) and orthopedic publications (H(2) = 22.86, *p* < 0.01, η^2 ^= 0.03) of students, excluding outliers (>95th percentile or number of total publications > 14), from a top 25 research school was greater than those of students from a non-top 25 research school. The horizontal line in each box indicates the median value; the top and bottom borders of the box show the 75th and 25th percentiles.

Our analysis showed that residents who completed their undergraduate medical training at a Top 25 medical school for research published significantly more articles overall (4.97 vs 3.51, *p* < 0.001, η^2 ^= 0.04), published significantly more orthopedic-related articles (3.90 vs 2.68, *p* <0.001, η^2 ^= 0.03), accrued significantly higher total AS (50.60 vs 24.20, *p* < 0.001, η^2 ^= 0.04) and average AS per article (8.85 vs 4.18, *p* < 0.001, η^2 ^= 0.04), and had a significantly higher h-index (2.76 vs 1.57, *p* <0.001, η^2 ^= 0.04) than those who graduated from non-Top-25 schools for research (Figure 2). Whether or not residents completed their undergraduate medical training at a top 25 school for primary care or had a home orthopedic department did not significantly influence the total number of publications, orthopedic publications, total AS, average AS, and h-index per resident.

## Discussion

Through our analysis, we identified significant differences in research productivity between residents from a medical school ranked in the top 25 for research and those who were not. We found that the total publication count, number of orthopedic publications, altmetric attention scores (both average and total), and h-indices of residents from the top 25 medical schools for research were higher than those from non-top 25 medical schools for research. Additionally, many residents did not have any publications while they were in medical school. On the other hand, there were at least 10 residents with more than 30 publications. At the same time, our effect sizes were less than 0.06, indicating that there are factors influencing an individual’s research productivity other than school research status alone. It’s unknown what the effects are when more demographic information such as race and ethnicity, data we didn’t have access to, are factored in.

What do our findings tell us about the aggregate match data published for ERAS?

Table 3 shows the mean research experiences and mean number of abstracts, presentations, and publications (APPs) for 2018, 2020, and 2022 [1,17,18]. While the mean number of APPs has noticeably risen, the mean number of research experiences has stayed relatively stagnant. Our results show that, on average, less than a quarter of the mean number of APPs for accepted applicants are publications. Most APPs are either abstracts or presentations. This suggests that the number of research experiences may not be as important for matching as the number of abstracts, presentations, and publications that come because of these experiences.

**Table 3:** Mean number of research experiences and abstracts, presentations, and publications (APPs) over the years

Year	Mean # Research Experiences of US Seniors Who Matched Into Ortho	Mean # Research Experiences of US Seniors Who Did Not Match Into Ortho	Mean # Abstracts, Presentations, and Publications of US Seniors Who Matched Into Ortho	Mean # Abstracts, Presentations, and Publications of US Seniors Who Did Not Match Into Ortho
2018	4.9	4.9	11.5	6.7
2020	5.4	5.7	14.3	14.2
2022	6.6	5.4	16.5	12.1

Additionally, there have been concerns about the reliance on the quantity versus quality of research activities in selecting desirable candidates. A study found that the number of publications for matched orthopedic surgery applicants was much lower than the reported national average of APPs [7]. Ngaage et al. suggest that while the number of APPs is increasing, the number of publications is not necessarily following that trend and discuss a shift in the types of research being published. None of this is evident from the aggregate numbers alone that many medical students and program directors rely on to inform themselves of an applicant’s readiness.

Another finding seen during data collection was the fact that a few residents who had over 30 publications took a research gap year (RGY) as confirmed by the research year program’s website or LinkedIn. This is far greater than the average number of APPs reported by the NRMP (Table 3). While these residents qualify as outliers, they represent a growing trend in orthopedic surgery and other competitive specialties [6,19]. In addition, we found that a few of these students matched at the institution they took their gap year with. Similar observations were found in a study done by Cotter et al. where 19.4% of RGY applicants who matched into orthopedics did so at the same institution they did their RGY. In addition, Cotter et al. show that applicants who took an RGY had more research activities than non-RGY applicants, but the number of research activities from their study was not found to be highly associated with match success. Research suggests that residency programs’ research productivity status influences how much interns publish [20]. The same can likely be said for the status of medical schools and the productivity of their students who apply for residency. One study found that higher-tier orthopedic programs accept applicants with greater research productivity [21]. They also looked at factors such as the number of publications, h-index, and citations. However, the study did not look at additional variables such as the applicant's medical school ranking or any social networking measures.

Are students with the hopes of matching into orthopedics engaging in scholarly activity through social networking?

To our knowledge, this is the first study that analyzes altmetric attention score of orthopedic surgery residency applicants. When assessing research impact, the use of the non-traditional altmetric attention score proves advantageous in its immediate availability, measurement of initial reactions towards publications, and longitudinal tracking of influence and attention over time [10]. An advantage of the altmetric attention score is that some research funders are looking at it to assess which research is receiving early engagement long before it would receive academic citations [22]. The results of our analysis do not suggest that orthopedic surgery applicants are utilizing social networks for academic research. Therefore, the use of the altmetric attention score specifically for orthopedics applicants may not currently be a fair or reliable measure for evaluating the research impact of students.

What is the best metric?

Currently, the number of APPs is being used to assess research productivity. The use of this number by programs is not inherently bad if combined with the understanding that the bulk of APPs are abstracts and presentations rather than publications for most applicants. Applicants should be aware that other students are not publishing as much as they might think. Additionally, while h-index can be considered, it lacks meaning for applicants without publications.

The altmetric attention score could be used more as more applicants increase their use of social media to disseminate their research findings. Other bibliographic metrics can take years to accumulate, while diffusion of publications on social media can happen much quicker and can be a better reflection of applicant publication impact for applications. While altmetric attention score can serve as a good indicator as to how much social interest a paper has generated, it should be interpreted with caution. Altmetric attention score measures the amount of social media attention a paper gets but it does not disclose information on the quality of a paper or differentiate between good attention versus bad attention. For example, if a paper receives a high volume of social media attention for being an inaccurate article, altmetric only sees that as having a high number of web-driven interactions regardless of reason. However, negative press is subjective in research as this could stimulate a productive discussion among readers because the paper is found to be controversial rather than inaccurate. Either way, this wouldn’t be clear from the number alone. Altmetric attention score is a metric that should be kept in mind for future use for more accurate analysis of applicants when dispersion and discussion of publications through social media becomes a more popular form of engagement in this field.

What is a possible consequence of an increased focus on publications in the wake of a pass/fail Step 1?

One possible consequence of an increased focus on publications is applicants from a non-top 25 school for research being disadvantaged when applying for orthopedic surgery residency. US News Report stated that two of the factors implemented when ranking the Top 25 Medical Schools for Research are "Faculty Resources" and "Average federal research activity per faculty member", which calculates research activity based on medical school financing data [15]. This means that the leading schools in research are the schools with more faculty, money, and resources at their disposal to conduct research. A stronger emphasis on research done in medical school could cause qualified applicants to be at risk of not matching simply because they did not have as many opportunities for projects as students from other institutions.

Limitations and future work

While our analysis focuses on the publication aspect of research productivity to better understand aggregate data, there are some limitations that deserve additional evaluation. First, we were restricted to resident publications only rather than residents’ full research activity. We understand that publications do not tell the full story of an applicant's research involvement. However, publications are more easily verifiable and are often promoted as being vital to matching while the aggregate APPs still don't give insight into the number of publications. Since only published manuscripts could be searched for, we could not account for submitted papers that had not yet been accepted or papers under revision. It is not unlikely that some applicants were unable to publish their projects in time for the residency application cycle. Additionally, despite many efforts to verify applicant identity and check various platforms to ensure our study analyzed the correct candidate and all their publications, there is no guarantee that all publications could be accounted for. Applicants may have undergone name changes during medical school. This would have affected the publication counts in our analysis, but it is unlikely that the overall trends would be significantly different. Also, our study was not able to evaluate the same data for applicants who applied to orthopedic surgery residencies and did not match. This data would have been beneficial in further assessing the value of publications to programs when determining which candidates to accept.

Lastly, another limitation of this study includes candidate self-inflation of altmetric attention scores through self-advertising on social media. This is similar to the limitation of self-inflation faced with using the h-index, where candidates can self-cite in subsequent publications. Additionally, the altmetric attention score is time-dependent and recalculated daily. This means that altmetric attention scores were possibly lower at the time of residency applications than when we collected altmetric attention scores. This depends on the publication date and the fluctuating attention each publication receives. If residency programs were to consider altmetric attention scores during future application cycles, it would be important for them to try to review the scores of all applicants' research within a shorter window of time to limit variability.

## Conclusions

In summary, our analysis of 688 orthopedic residents showed that residents from the top 25 medical schools for research had more research publications, higher average altmetric attention scores, and higher h-indices than those from non-top 25 medical schools for research. Our findings give insight to future orthopedic surgery applicants as to the different research parameters residency programs can examine when evaluating applicants and what the average applicant looks like. This can help guide these applicants on ways to demonstrate their interest in orthopedics throughout the medical school while informing them that other applicants are not publishing as much as they may think. Students should continue to be encouraged to not overlook the quality of their research projects, and programs should be increasingly encouraged to consider the quality of applicant research and an applicant's research setting. Additionally, the altmetric attention score may not yet be the best way of qualifying research experience. However, it has the potential to be a useful metric if orthopedic surgery applicants increase their use of social media as a way of disseminating their scientific findings.
